# The complete mitochondrial genome of *Exostoma gaoligongense* (Siluriformes: Sisoridae) and its phylogenetic analysis within glyptosternine catfishes

**DOI:** 10.1080/23802359.2021.1912670

**Published:** 2021-04-26

**Authors:** Zheng Gong, Feng Lin, Zunlan Luo, Xiaoyong Chen

**Affiliations:** a College of Life Sciences, Zaozhuang University, Zaozhuang, China; b University of Chinese Academy of Sciences, Beijing, China; c Kunming Institute of Zoology, Chinese Academy of Sciences, Kunming, China; d Southeast Asia Biodiversity Research Institute, Chinese Academy of Sciences, Nay Pyi Taw, Myanmar; e Chinese Research Academy of Environmental Sciences, Beijing, China

**Keywords:** *Exostoma gaoligongense*, mitochondrial genome, phylogenetic analysis

## Abstract

*Exostoma gaoligongense* is an endemic glyptosternine catfish distributed in the Nujiang River drainage, Yunnan Province, China, with few published genetic information. In this study, we sequenced and characterized the complete mitochondrial genome of *E. gaoligongense*, which was circular, 16529 bp in length, containing 13 protein-coding genes, 22 tRNA genes, 2 rRNA genes, one replication origin and one control region. Phylogenetic analysis revealed that *E. gaoligongense* had the closest relationship with its congener *E. labiatum*. They clustered with the clade containing most genera of glyptosternine catfishes and then cluster with the more primitive genera *Glaridoglanis* and *Glyptosternon*.

The genus *Exostoma* Blyth 1860, belonging to the glyptosternine catfishes within Sisoridae, is characterized by having a strongly depressed body profiles and horizontally enlarged paired fins modified for adhesion, continuous post-labial groove, homodont dentition consisting of distally flattened oar-shaped teeth and two narrowly-separated patches on the upper jaw and two well-separated patches on the lower jaw (Thomson and Page 2006; Darshan et al. 2019). Species of genus *Exostoma* primarily inhabit the bottom of the rocks in the swift-flowing mountain streams or rivers from the Yarlung Tsangpo-Brahmaputra River drainage eastwards to the Lancang-Mekong River drainage (Gong et al. 2018). To date, 18 valid species within this genus have been described (Luo and Chen 2020; Arunkumar 2020). *E. gaoligongense*, recently described by Chen et al. (2017) based on morphological diagnosis, was endemic to four tributaries (Manggang River, Tangxi River, Xinya River and Nanzha River) of Nujiang (upper Salween) River in Yunnan, China.

In this study, a female specimen of *E. gaoligongense* (52 mm standard length) was collected during June, 2020 from Kungong River, a tributary of Nujiang River in Lujiang Town, Baoshan City, Yunnan (new record of distribution, 25°5′40.9″N, 98°49′18.6″E, 797 m), which were deposited at the Kunming Natural History Museum of Zoology, Chinese Academy of Sciences (voucher number: KIZ 2020000021). The genome DNA was extracted from a bit of fin tissue using the Animal Tissue DNA Extraction Kit (TSP201, Tsingke, China). Paired-end sequencing for the mitogenome of *E. gaoligongense* was performed on the Illumina Hiseq platform (Illumina Inc., San Diego, CA). The obtained raw sequence reads were assembled into contigs using SPAdes 3.9 (Bankevich et al. 2012) and the annotation was conducted in MITOS (Bernt et al. 2013).

The complete mitogenome sequence of *E. gaoligongense* is circular and 16529 bp in length, including 13 protein-coding genes, 22 tRNA genes, two rRNA genes, one replication origin (O_L_) and one control region (D-loop). The total nucleotide composition of *E. gaoligongense* mitogenome is 32.4% A, 28.7% C, 24.3% T, and 14.6% G. The high A-T content pattern is consistent with the typical feature of the vertebrate mitochondrial genome (Mayfield and McKenna 1978). Most of the protein-coding genes started with ATG as the initiation codon except for *CO1* gene starting with GTG. *ND4*, *COX2*, *COX3*, and *Cytb* genes ended with T- whereas the remaining 9 protein-coding genes ended with ATR (R represents A or G). Structure of protein-coding genes in *E. gaoligongense* was identical to that in its congener *E. labiatum* (NC021601).

The 13 mitochondrial protein-coding genes were combined from *E. gaoligongense* and 16 additional species including 15 glyptosternine species and one outgroup (*Pseudecheneis sulcatus*) for phylogenetic analysis. Sequence alignment was performed using MEGA X 10.0 (Kumar et al. 2018). Nucleotide substitution model (GTR + I + G) was chosen based on Akaike information criterion (AIC) using jModelTest 2.1 (Darriba et al. 2012). Phylogenetic analysis was performed based on Bayesian inference in MrBayes 3.1 (Ronquist and Huelsenbeck 2003).

The topology of the phylogenetic tree showed that *E. gaoligongense* clustered with its congener *E. labiatum* (NC021601, location: Nujiang River) with small genetic divergence of 1.6% Kimura 2-parameter distance. In addition, *Exostoma* species clustered with the clade containing the most genera of glyptosternine catfishes and then clustered with the more primitive groups *Glaridoglanis* and *Glyptosternon* (Figure 1). The mitogenome announcement of *E. gaoligongense* could help understand the phylogenetic position of this genus, as well as offer valuable genetic information for further study on the genetic diversity and biological conservation of this species.

**Figure 1.**
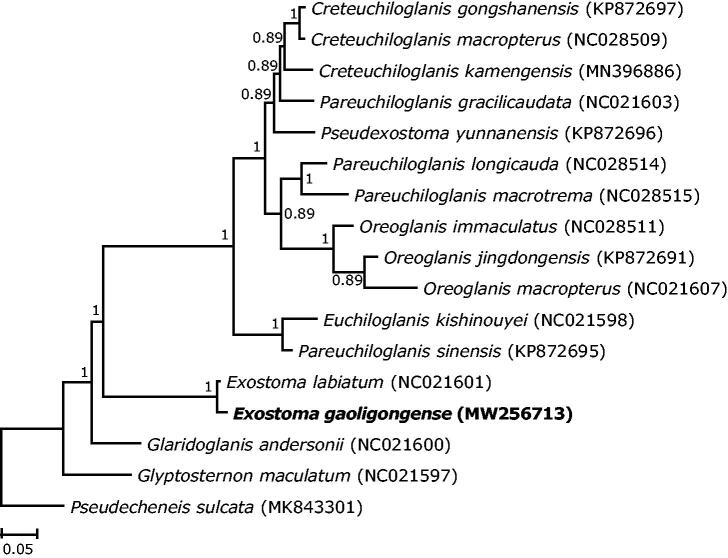
Phylogenetic tree of 16 glyptosternine catfishes based on Bayesian inference of 13 protein-coding genes. The posterior probabilities were shown on the nodes. The GenBank accession numbers of included species were shown in brackets.

## Discloser statement

All authors declare no potential conflict of interest.

## Data Availability

Mitogenome data supporting this study are openly available in GenBank at https://www.ncbi.nlm.nih.gov/nuccore/1945652357.
